# UPLC-QTOF-MS Identification of the Chemical Constituents in Rat Plasma and Urine after Oral Administration of *Rubia cordifolia* L. Extract

**DOI:** 10.3390/molecules22081327

**Published:** 2017-08-11

**Authors:** Zuoliang Zheng, Shengqing Li, Yuping Zhong, Ruoting Zhan, Yan Yan, Huafeng Pan, Ping Yan

**Affiliations:** 1 Research Center of Chinese Herbal Resource Science and Engineering, Guangzhou University of Traditional Chinese Medicine, Guangzhou 510006, China; 15975502279@sina.cn (Z.Z.); ruotingzhan@vip.163.com (R.Z.); 2 Key Laboratory of Chinese Medicinal Resources from Lingnan of Ministry of Education, Joint Laboratory of National Engineering Research Center for the Pharmaceutics of Traditional Chinese Medicines, Guangzhou University of Traditional Chinese Medicine, Guangzhou 510006, China; 3 Institute of Traditional Chinese Medicine, Guangzhou University of Traditional Chinese Medicine, Guangzhou 510006, China; gzzyylsq@sina.com (S.L.); 15914272434@sina.cn (Y.Z.); gzzyydxyy@sina.com (Y.Y.); 4 Guangzhou University of Traditional Chinese Medicine, Guangzhou 510006, China

**Keywords:** *Rubia cordifolia* L., UPLC/Q-TOF-MS, metabolites, alizarin, purpurin

## Abstract

An effective ultra-performance liquid chromatography coupled with the quadrupole time-of-flight tandem mass spectrometry (UPLC/Q-TOF/MS) method was developed for analysing the chemical constituents in rat plasma and urine after the oral administration of *Rubia cordifolia* L. extract. Under the optimized conditions, nine of 11 prototypes in rat plasma and four prototypes in urine were identified or characterized by comparing the retention time, accurate mass, fragmentation patterns, reference compounds, and literature data. In total, six metabolites, including alizarin-1-*O*-β-glucuronide, alizarin-2-*O*-β-glucuronide, alizarin-1-*O*-sulfation, alizarin-2-*O*-sulfation, purpurin-1-*O*-β-glucuronide, and purpurin-3-*O*-β-glucuronide, were identified in rat plasma, which were confirmed by lavaging standard solutions. Purpurin was found to be able to be transformed into alizarin based on the results in which alizarin was detected in rat plasma after the oral administration of a purpurin solution. In total, four metabolites were found in rat urine, but their chemical structures were not confirmed. The results indicate that the metabolic pathway of alizarin involves glucuronidation and sulfation, with the purpurins having undergone glucuronidation. The components absorbed into the blood, and the metabolites have the opportunity to become bioactive constituents. The experimental results would supply a helpful chemical basis for further research on the mechanism of actions of *Rubia cordifolia* L.

## 1. Introduction

*Rubia cordifolia* L. was officially listed in the 2015 edition of the Chinese Pharmacopoeia and is widely used as a traditional Chinese medicine for the treatment of tuberculosis, contusions, menoxenia, and rheumatism in China, Japan, Korea, and India [1]. Many constituents, including anthraquinones, naphthoquinones, naphthodroquinones, triterpenes [2,3], and iridoids [4,5] were isolated and identified from the genus *Rubia*. To the best of our knowledge, different parts of *Rubia cordifolia* L. have different chemical compositions. However, ony the bioactive components that are detected in the blood could exert therapeutic effects and contribute to the quality of traditional Chinese herbal medicines (TCMs) [6]. In the previous literature, several anthraquinones and anthraquinone derivatives were proven to exert different pharmacological activities [7], including antifungal, antioxidant, antimicrobial, anti-inflammation, antibacterial, and anticancer activities [8,9,10,11,12]. Purpurin showed stronger antioxidant and better enzyme inhibitory effects than mollugin [13], which had a good reputation for its anti-carcinogenic and anti-viral activities [14]. In addition, pseudopurpurin could increase the bone mineral density and enhance the geometry of its architecture [15]. Rubiadin exhibits a potent hepatoprotective action against carbon tetrachloride-induced hepatic damage in rats [16].

The research on drug bioactivities is based on the drug metabolism. Therefore, the identification of drug prototypes and metabolites in vitro or in vivo is of vital importance for elucidating drug pharmacological mechanisms and pharmacokinetic behavior. Anthraquinones and anthraquinone derivatives have been proved to be the main active substance in *Rubia cordifolia* L. acting in disease. However, its prototypes and metabolites in vivo have not been reported. Nowadays, it is difficult to identify them due to interference from endogenous metabolites, their extremely low concentrations [17], a lack of standards, and diverse structure types. Their many isomers further increased the difficulty of the analysis and the uncertainty of the results. Therefore, the ultra-performance liquid chromatography coupled with the quadrupole time-of-flight tandem mass spectrometry (UPLC/Q-TOF-MS) has been proved to be an effective analytical tool for the determination of chemical constituents and metabolites in biological samples by means of its selectivity, sensitivity, and speediness [18,19,20]. In this paper, a UPLC/Q-TOF-MS method was developed to systematically analyze the prototypes and metabolites in rat plasma and urine after oral administration of *Rubia cordifolia* L. for revealing the metabolic pathways and understanding the mechanism of action of *Rubia cordifolia* L.

## 2. Results and Discussion

### 2.1. Identification of Chemical Components in Rubia cordifolia L. Extract

**Figure 1:**
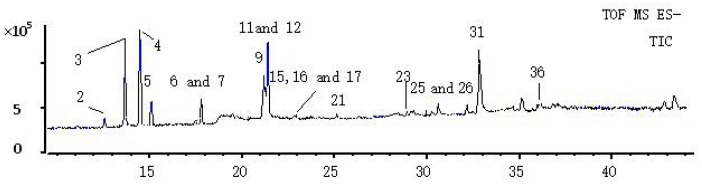
TIC of *Rubia cordifolia* L. extract.

The identification of the chemical constituents in the plasma and urine was based on the analysis of the components of the *Rubia cordifolia* L. extract. The information from the 40 compounds in *Rubia cordifolia* L. extract, including the chromatogram and MS^2^ spectra, was recorded (Table 1). Compounds **23**, **25**, **26**, **31**, and **32** were confirmed to be 6-hydroxyrubiadin, alizarin, purpurin, physcion and rubiadin, respectively, by comparison with the reference compounds. A total of nine compounds were tentatively characterized based on the comparison of literature data and the analysis of the fragmentation regularities using the PeakView (Version 2.0, AB SCIEX) [15,21,22]. The remaining 26 ingredients were not further analyzed due to the lack of authentic compounds and reference data. The profiling of these compounds was shown by a total ion chromatogram (TIC) in Figure 1.

**Figure 2:**
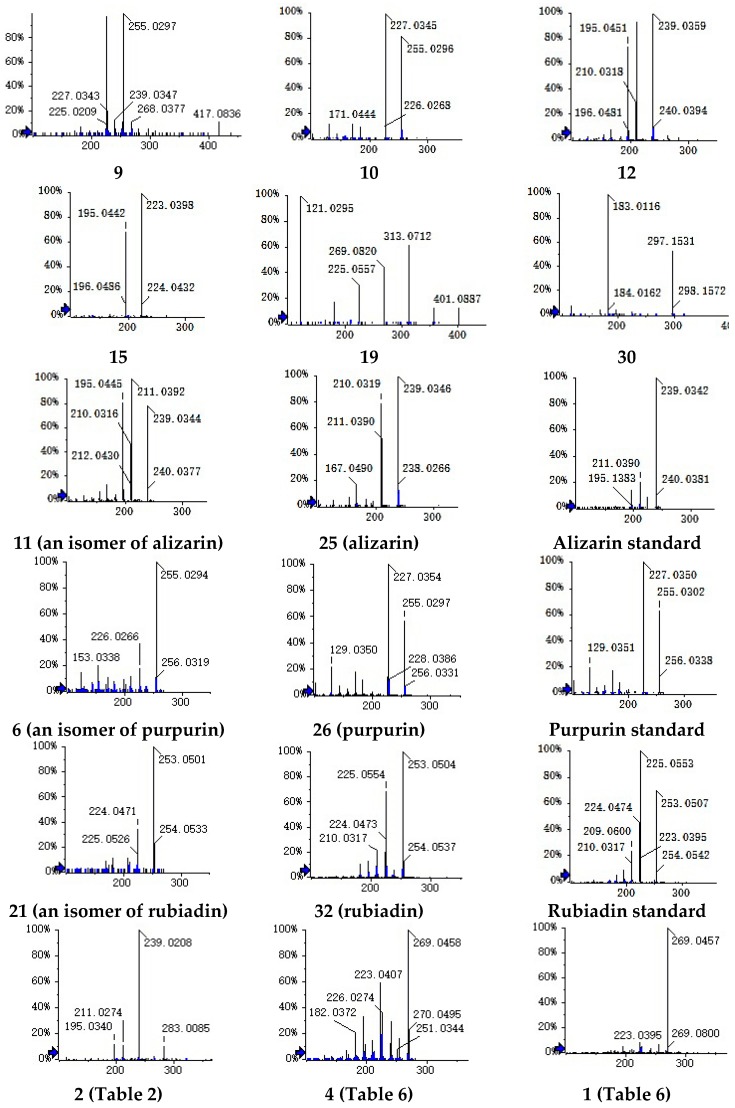
Spectra of compounds.

Compound **10** (Table 1) presented the parent ion at a *m*/*z* of 299.0201 ([M − H]^−^, 0.6 ppm). The product ions at the *m*/*z* of 255 contributed to the elimination of CO_2_. The fragment ions (227, 183, 171, 143, and 129) that were observed in the MS^2^ spectra (Figure 2) were in accord with the purpurins (compound **26**; Figure 2). Thus, the compound **10** was identified as pseudopurpurin.

Compounds **11** and **25** at retention times of 21.41 and 30.56 min shared the same precursor ion at the *m*/*z* of 239.0346 and recorded the coincident fragmentation pathways. Therefore, they were considered isomers. Compound **25** had been determined as alizarin (Figure 2). Thus, the compounds **6** (Table 2) and **21** were tentatively identified as isomers of purpurin and rubiadin, respectively.

Based on the molecular weight, fragmentation ions, and previously published data [21], the compounds **9**, **12**, **15**, **19**, and **30** were tentatively confirmed as 2-methyl-1,3,6-hydroxy-9,10-anthraquinone-3-*O*-β-d-glucopyranoside, munjistin, nordamnacanthal, alizarin-2-*O*-Glc, and 1-hydroxy-2-carboxyl-3-methoxy-anthraquinone, respectively. The MS^2^ spectra of compounds **9**, **11**, **12**, **15**, **19**, **21**, **25**, and **32** are presented in Figure 2.

### 2.2. Detection of the Prototype Components and Metabolites in Rat Plasma

**Figure 3:**
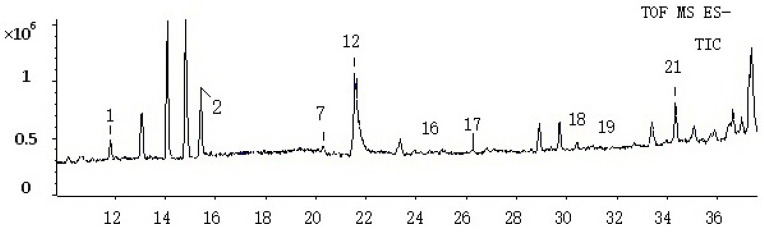

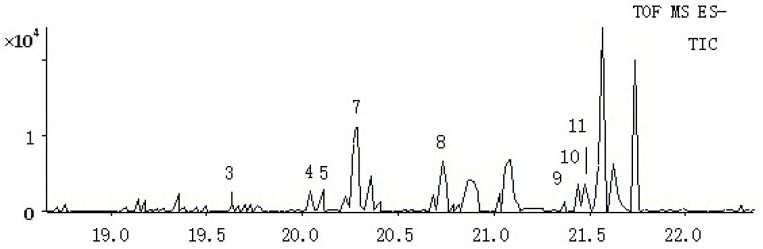
TIC of the rat plasma sample.

After eliminating the interference of endogenous substances, 21 compounds (including 11 prototype components and 10 metabolites) were found, but only 13 of these components were tentatively identified (Figure 3).

As shown in Table 1 and Table 2, the compounds **10**, **11**, **12**, **15**, **17**, **19**, **21**, **25**, **26**, **27**, and **32** in the *Rubia cordifolia* L. extract correspond to the compounds **9**, **10**, **11**, **14**, **15**, **16**, **17**, **18**, **19**, **20**, and **21** in the rat plasma, respectively. It was concluded that these compounds can be regarded as prototype components.

According to Table 2, compound **2** shows a parent ion at a *m*/*z* of 459.0562 and product ions at the *m*/*z* of 283, 239, 211, and 195. The product ion at a *m*/*z* of 283 was 176 Da less than the deprotonated molecule. Based on the chemical constituents of *Rubia cordifolia* L., the fragment at the *m*/*z* of 283 represents the structure of munjistin. Thus, it was concluded that compound **2** was generated through the glucuronide conjugation of munjistin. Therefore, compounds **4** and **5** were identified as glucuronide conjugations of alizarin, while compounds **3** and **7** were confirmed to be the glucuronide conjugation of purpurin. The MS^2^ spectrum of compound **2** is shown in Figure 2. The parent ions of compounds **8** and **13** at the *m*/*z* of 319 are 80 Da larger than alizarin. Thus, they were identified as sulfation products of alizarin.

**Figure 4:**
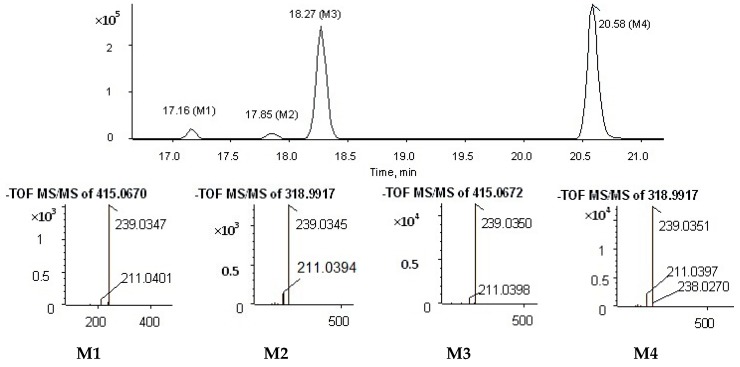
Metabolite chromatogram of alizarin.

**Figure 5:**
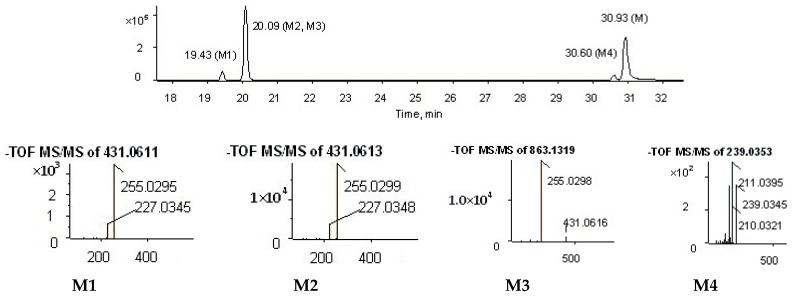
Metabolite chromatogram of purpurin.

**Figure 6:**
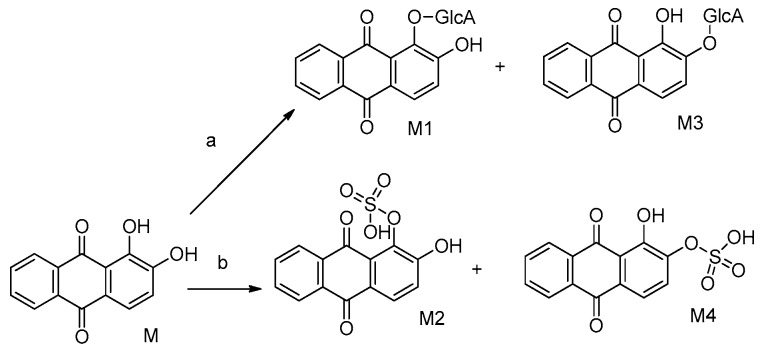
Metabolic pathways of alizarin.

**Figure 7:**
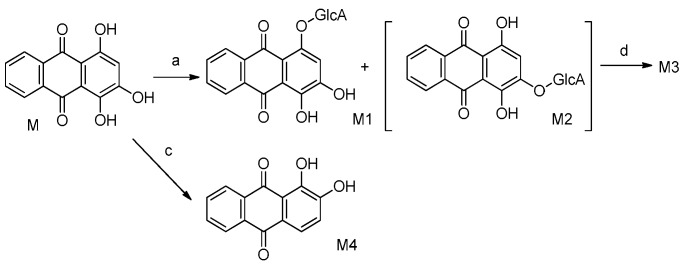
Metabolic pathways of purpurin. Metabolic pathways: (**a**) glucuronidation; (**b**) sulfation; (**c**,**d**) unknown.

The metabolites of alizarin and purpurin metabolites were observed in rat plasma after the administration of an alizarin solution and a purpurin solution, respectively. Figure 4 and Figure 5 obtained using Metabolite Pilot 1.5 software show the metabolite chromatograms of alizarin and purpurin, respectively. The information for the metabolites are shown in Table 3 and Table 4. M1 and M3 were identified as alizarin-1-*O*-β-glucuronide and alizarin-2-*O*-β-glucuronide, based on their same molecular weight, different retention times, and different peak areas (the peak area of M3 > M1), because the β-*O*H of antraquinones is more active and the result is consistent with the study of emodin [23]. Therefore, M2 and M4 were similarly confirmed to be alizarin-1-*O*-sulfation and alizarin-2-*O*-sulfation, respectively. According to the Table 4 and the MS^2^ spectra (shown in Figure 5), M1 and M2 in the metabolite chromatogram of purpurin were identified as purpurin-1-*O*-β-glucuronide and purpurin-3-*O*-β-glucuronide, respectively, while M4 was regarded as alizarin. The interference of alizarin in purpurin standard can be excluded due to the purity of purpurin standard and the peak area of alizarin (shown in Table 4). The MS^2^ spectrum of M4 can be found in Figure 5. Therefore, it is concluded that purpurin could transform into alizarin in the rat body. The result is consistent with the study of emodin that stated that emodin could transform into chrysophanol [24]. The M3 was not determined, but it was deduced to be a metabolite of M2 due to their same fragment ions at the *m*/*z* of 431, 255, and 227. The metabolic pathways of alizarin and purpurin are shown in Figure 6 and Figure 7, respectively. The chemical structures of the metabolites and the identified compounds are summarized in Table 5. The glucuronic acid (GlcA) and sulfuric acid ester group binding sites in the anthraquinones are α-*O*H and β-*O*H. As shown in Table 2, the compounds **1**, **2**, **6**, and **12** were found as metabolites. However, further studies are needed to reveal the structures of these compounds.

### 2.3. Detection of the Chemical Constituents in Rat Urine

**Figure 8:**
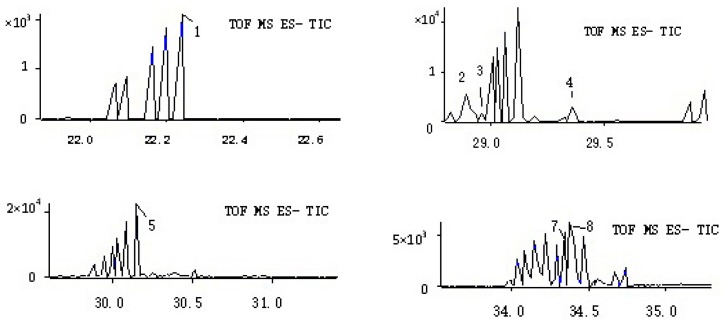
TIC of the rat urine sample.

It could be observed from Table 6 that eight compounds were detected in rat urine. Among them, the compounds **1**, **2**, **3**, and **8** were regarded as metabolites. In comparison, the other compounds were regarded as prototypes. However, most of the chemical constituents were not identified. The TIC was shown in Figure 8.

The parent ion of Compound **1** at a *m*/*z* of 349.0031 was 80 Da more than the precursor ion of compound **4** (6-Hydroxyrubiadin). It was regarded as the sulfation product of 6-Hydroxyrubiadin, based on the MS^2^ spectra (Figure 2). The 6-hydroxyrubiadin was not detected in the rat plasma but it was found in the urine, suggesting that it may not be a bioactive constituent.

## 3. Materials and Methods

### 3.1. Chemcials, Reagents, and Meterials

*Rubia cordifolia* L. were purchased from Guangdong Medicinal Materials and Yin Pian Company (Guangzhou, China), and were further identified by Professor Ruo-Ting Zhan (Guangzhou University of Traditional Chinese Medicine, Guangzhou, China). The reference standards of alizarin, purpurin, and physcion (purity of >98%) were provided by the China Institute of Pharmaceutical and Miological products, while the 6-pydroxyrubiadin and rubiadin (purity of >98%) were obtained from BioBioPha. HPLC-grade methanol, acetonitrile, and formic acid were purchased from Merck (Merck, Darmstadt, Germany). Purified water was prepared from a Milli-Q system (Millipore Billerica, MA, USA).

### 3.2. Instrument and Analytical Conditions

Chromatographic separation was performed by a Venusil XBP (L) C18 column (4.6 × 250 mm, 5 μm; Agela) at 30 °C. Several mobile phase systems, including methanol-water, acetonitrile-water, acetic acid (0.1% and 0.05%), and formic acid (0.1% and 0.05%) were tested to identify the optimal mobile phase. Ultimately, a mobile phase of acetonitrile (A) and 0.05% formic acid-water (B) was selected. The gradient program was as follows: 0–10 min at 10–25% A; 10–25 min at 25–50% A; 25–35 min at 50–75% A; 35–45 min at 75% A; 45–55 min at 75–100% A; and 55–60 min at 100% A. The mobile phase rate was 0.8 mL/min and each injection volume was set at 10 μL.

The MS data were acquired on an AB SCIEX Triple TOF 5600 (AB sciex Pte. Ltd., Singapore). The Mass spectrometric parameters were as follows: interface of negative electrospray ionization (ESI); gas 1 and 2 being nitrogen 55 psi; curtain gas being nitrogen 40 psi; source temperature of 400 °C; ion spray voltage of 5500 V; de-clustering potential of 100 V and collision energy of 45 eV. The Peakview (Version 2.0, AB SCIEX) and MetabolitePilot^TM^ (Version 1.5, AB SCIEX) were employed for the analyses.

### 3.3. Animals, Dosage, and Biological Sample Collection

Eighteen male Sprague-Dawley rats (weight of 180–220 g) were provided by the Experimental Animal Center of the Guangzhou University of Chinese Medicine and randomly divided into three groups (Group I, Group II, and Group III) of six rats each. These animals were housed in a breeding room at a controlled temperature (20–24 °C) and humidity (40%–60%) in a 12 h light/dark cycle with free access to food and water for three days. All rats were fasted for 12 h with free access to water prior to the experiment.

*Rubia cordifolia* L. was heating by being extracted with 70% ethanol for 1 h and then filtered. The moisture in the filtrate was evaporated and the residue was dissolved in methanol to a concentration equivalent to 1 mg/mL of the *Rubia cordifolia* L. for analysis.

*Rubia cordifolia* L. was immersed in 70% ethanol (1:8, *w*/*v*) and extracted three times (1 h each time). The extracted solutions were combined and concentrated under a reduced pressure to a density of 1 g/mL for oral administration.

Alizarin solution (0.63 mg/mL) and purpurin solution (3.25 mg/mL) were each prepared with 0.5% aqueous CMCC-Na for oral administration.

The *Rubia cordifolia* L. solution (1 g/mL), the alizarin solution (0.63 mg/mL), and the purpurin solution (3.25 mg/mL) were orally administered to group I (10 g/kg body weight), group II (6.3 mg/kg body weight), and group III (32.5 mg/kg body weight), respectively. Blank blood samples and medicated blood samples were collected from the suborbital vein before administration and 2 h after administration, respectively. Following this, these samples were and then immediately centrifuged for 5 min at 14,000 rpm at 4 °C.

All urine samples from group I were collected for 12 h post-dosing and combined into one sample for the purpose of eliminating the individual variability.

### 3.4. Biological Sample Preparation

The supernatants of the two types of blood samples from the same group were mixed into a single sample to eliminate individual interference. A total of 200 μL aliquots of mixed plasma samples were precipitated with 800 μL methanol and vortexed for 5 min. The sample was centrifuged at 14,000 rpm for 5 min, and beforehand the supernatant was separated and dried under a stream of nitrogen gas at 30 °C. The residue was dissolved in 200 μL of methanol, before 10 μL of this mixture was injected into the UPLC/Q-TOF/ MS for analysis.

The mixed urine sample was homogenized with methanol at a ratio of 1:4, before being vortexed and centrifuged at 14,000 rpm for 5 min. Following this, the supernatant was removed and evaporated to dryness. The residue was dissolved in 200 μL of methanol for UPLC/Q-TOF MS analysis.

## 4. Conclusions

In this study, a UPLC/Q-TOF-MS method was established for studying the metabolism of *Rubia cordifolia* L. Eventually, nine prototype components and six metabolites including alizarin-1-*O*-β-glucuronide, alizarin-2-*O*-β-glucuronide, alizarin-1-*O*-sulfation, alizarin-2-*O*-sulfation, purpurin-1-*O*-β-glucuronide, and purpurin-3-*O*-β-glucuronide were identified in the plasma. Also, this indicates that the metabolic pathway of alizarin involves glucuronidation and sulfation, with the purpurins having undergone glucuronidation. Thus, *Rubia cordifolia* L. possibly express its effects through their metabolites. Additionally, it is interesting that purpurin can transform into alizarin in rat body. Research has made it clear that alizarin and purpurin both can induce gene mutations that contribute to cases of nephrotoxicity [25,26]. Thus, it is hypothesized that alizarin, instead of purpurin, is associated with the noxious effects to kidney. The analysis of the changes in purpurin content in the body may be the best way to understand the metabolic pathways of purpurin. Furthermore, four prototype components were identified in urine. The 6-pydroxyrubiadin was only detected in the urine, suggesting that it may not be the active substance of *Rubia cordifolia* L. However, further studies based on the nuclear magnetic resonance technology (NMR) are needed to identify the unidentified compounds. This experiment might provide a basis for further pharmacological and pharmacokinetic research on *Rubia cordifolia* L.

## Figures and Tables

**Table 1:** Compounds identified in *Rubia cordifolia* L. extract.

NO.	RT	Mass Found	Error	MS^2^ Ions	Identification
	(min)		(ppm)		
**1**	10.68	461.0732	1.2	417, 298, 280, 255,226	
**2**	12.55	610.4194	0.1	564, 546, 451, 338, 225, 130	
**3**	13.7	723.5057	0	677, 659, 564, 451, 338, 225	
**4**	14.52	836.5906	0.3	790, 225	
**5**	15.15	949.6771	0.2	903	
**6**	17.62	563.1419	0.2	269, 240	Ruberythric acid
**7**	17.86	577.1579	0.2	577, 269, 240	
**8**	19.57	619.1667	0.9	577, 559, 269, 240	
**9**	20.06	417.0836	1.5	255, 241	2-Methyl-1,3,6-hydroxy-9,10-anthraquinone-3-*O*-β-d-glucopyranoside
**10**	21.32	299.0201	0.6	255, 227, 183, 171, 143, 129	Pseudopurpurin
**11**	21.41	239.0346	1.1	211, 195, 183, 167, 155	An isomer of alizarin
**12**	21.43	283.0254	0.4	239, 211, 195, 167	Munjistin
**13**	21.59	619.1683	0	577, 269, 240
**14**	21.61	665.1745	0.1	619, 577, 372, 269, 239
**15**	23.05	267.0304	0.6	223, 195	Nordamnacanthal
**16**	23.1	473.1093	0.6	268, 240
**17**	23.12	345.0408	0.5	317, 301, 289, 273, 260, 245	Unknown
**18**	23.93	661.1793	0.4	619, 601, 269, 240
**19**	24.4	401.0887	0.3	356, 328, 300, 272, 244	Alizarin-2-*O*-Glc
**20**	25.37	801.3497	0.1	755, 630, 556, 493, 460
**21**	26.23	253.0504	0.1	225, 209, 195	An isomer of rubiadin
**22**	28.42	453.0623	0	409, 394, 350, 306, 293
**23**	29.53	269.0461	0.5	254, 241, 223, 210, 195	6-Hydroxyrubiadin
**24**	28.45	317.1033	0	213, 185, 157, 129
**25**	30.56	239.0343	0.6	211, 195, 167, 155	Alizarin
**26**	30.9	255.0297	0	227, 183, 171, 143, 129, 101	Purpurin
**27**	30.91	293.1764	0.1	236, 221, 205, 192, 177
**28**	30.99	593.1315	0.3	549, 505, 417, 383, 357, 313
**29**	31.15	745.2326	0.1	644, 513, 496, 482
**30**	31.63	297.1531	1	269, 254, 239, 223, 211, 197, 183, 169	1-Hydroxy-2-carboxyl-3-methoxy-anthraquinone
**31**	33.74	283.0612	2.3	268, 240, 211	Physcion
**32**	34.32	253.0502	0.5	225, 209, 195	Rubiadin
**33**	34.77	297.0773	0.5	251, 223, 195
**34**	34.93	457.0722	0.4	413, 384, 369, 356
**35**	36.28	441.135	0.1	372, 313, 297, 269
**36**	36.42	313.0511	0.1	285, 269, 257, 229, 201
**37**	37.7	325.1845	0.7	279, 197, 183, 119
**38**	38.36	295.228	1	277, 259, 183, 171
**39**	38.52	339.2003	0.3	197, 183, 119
**40**	43.24	353.2123	0	177, 163

**Table 2:** Compounds identified in rat plasma after oral administration of *Rubia cordifolia* L. extract.

NO.	RT (min)	Mass Found	Error (ppm)	MS^2^ Ions	Identification
**1**	11.8	497.3337	0	451, 433, 225	
**2**	15.34	459.0562	0.2	283, 239, 211, 195	Glucuronide of munjistin
**3**	19.64	431.0611	0.6	255, 227, 183	Purpurin-1-*O*-β-glucuronide
**4**	20.04	415.0670	0.3	239, 211, 167	Alizarin-1-*O*-β-glucuronide
**5**	20.09	415.0672	0.2	239, 211, 167	Alizarin-2-*O*-β-glucuronide
**6**	20.09	255.0298	0.1	255, 227, 183, 171	An isomer of purpurin
**7**	20.28	431.0613	1.2	255, 227	Purpurin-3-*O*-β-glucuronide
**8**	20.79	318.9917	0.4	239, 211, 183, 167, 155	Alizarin-1-*O*-sulfation
**9**	21.32	299.0201	0.6	255, 227, 183, 171, 143	Pseudopurpurin
**10**	21.41	239.0346	1.1	211, 195, 183, 167, 155	An isomer of alizarin
**11**	21.43	283.0254	0.4	239, 211, 195, 167	Munjistin
**12**	22.6	667.1307	0.2	491, 315	Glucuronide of compound 27 in Table 1
**13**	22.89	318.9917	0.3	239, 211, 183, 167, 155	Alizarin-2-*O*-sulfation
**14**	23.05	267.0304	0.6	223, 195	Nordamnacanthal
**15**	23.12	345.0408	0.5	317, 301, 289, 273, 260	
**16**	24.4	401.0879	0.3	356, 328, 300, 272, 244	Alizarin-2-*O*-Glc
**17**	26.23	253.0504	0.1	225, 209, 195	An isomer of rubiadin
**18**	30.56	239.0343	0.6	211, 195, 167, 155	Alizarin
**19**	30.9	255.0297	0	227, 183, 171, 143, 129	Purpurin
**20**	30.91	293.1764	0.1	236, 221, 205, 192, 177	
**21**	34.32	253.0502	0.5	225, 209, 195	Rubiadin

**Table 3:** Information on the metabolites of alizarin.

Peak ID	Formula	*m*/*z*	ppm	RT (min)	Peak Area	% Score
M1	C_20_H_16_O_10_	415.0670	0.3	17.16	1.19 × 10^6^	94.5
M2	C_14_H_8_O_7_S	318.9917	0.4	17.85	8.44 × 10^4^	95.0
M3	C_20_H_16_O_10_	415.0672	0.2	18.27	1.52 × 10^6^	96.9
M4	C_14_H_8_O_7_S	318.9917	0.3	20.58	1.89 × 10^6^	96.9

**Table 4:** Information on the metabolites of purpurin.

Peak ID	Formula	*m*/*z*	ppm	RT (min)	Peak Area	% Score
M1	C_20_H_16_O_11_	431.0611	0.6	19.43	3.20 × 10^5^	95.6
M2	C_20_H_16_O_11_	431.0613	1.2	20.09	2.19 × 10^6^	96.9
M3	Unknown	863.1319	0.0	20.09	9.60 × 10^5^	60.8
M4	C_14_H_8_O_4_	239.0346	1.2	30.60	2.35 × 10^5^	88.3
M	C_14_H_8_O_5_	255.0301	0.1	20.09	2.04 × 10^5^	91.0

**Table 5:**
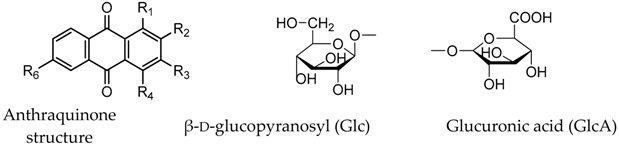
Chemical structures of some of the chemical constituents. Anthraquinone structure β-d-glucopyranosyl (Glc) Glucuronic acid (GlcA).

No.	Compound	Chemical Formula	Substituent Position				
			R_1_	R_2_	R_3_	R_4_	R_6_
**1**	Alizarin-1-*O*-β-glucuronide	C_20_H_16_O_10_	GlcA	OH	H	H	H
**2**	Alizarin-1-*O*-sulfation	C_14_H_7_O_4_SO_3_H	OSO_3_H	OH	H	H	H
**3**	Alizarin-2-*O*-β-glucuronide	C_20_H_16_O_10_	OH	GlcA	H	H	H
**4**	Purpurin-1-*O*-β-glucuronide	C_20_H_16_O_11_	GlcA	H	OH	OH	H
**5**	2-Methyl-1,3,6-hydroxy-9,10-anthraquinone 3-*O*-β-d-glucopyranoside	C_21_H_22_O_9_	OH	CH3	Glc	H	OH
**6**	Purpurin-3-*O*-β-glucuronide	C_2_0H_16_O_11_	OH	H	GlcA	OH	H
**7**	Alizarin-2-*O*-sulfation	C_14_H_7_O_4_SO_3_H	OH	OSO_3_H	H	H	H
**8**	Pseudopurpurin	C_15_H_8_O_7_	OH	OH	COOH	OH	H
**9**	Munjistin	C_15_H_8_O_6_	OH	COOH	OH	H	H
**10**	Nordamnacanthal	C_15_H_8_O_5_	H	COOH	H	OH	H
**11**	Alizarin-2-*O*-Glc	C_20_H_18_O_9_	OH	Glc	H	H	H
**12**	6-Hydroxyrubiadin	C_15_H_10_O_4_	OH	CH_3_	OH	H	H
**13**	Alizarin	C_14_H_8_O_4_	OH	OH	H	H	H
**14**	Purpurin	C_14_H_8_O_5_	OH	H	OH	OH	H
**15**	Rubiadin	C_15_H_10_O_4_	OH	CH_3_	OH	H	H

**Table 6:** Compounds identified in rat urine after oral administration of *Rubia cordifolia* L. extract.

NO.	RT	Mass Found	Error	MS^2^ Ions	Identification
	(min)		(ppm)		
**1**	22.24	349.0031	0.2	269, 254, 226	Sulfation of 6-Hydroxyrubiadin
**2**	28.89	254.0471	0.3	226, 183	
**3**	28.96	270.0505	0.1	255, 242, 227, 196	
**4**	29.53	269.0461	0.5	254, 241, 223, 195	6-Hydroxyrubiadin
**5**	30.12	239.0350	0.6	211, 195, 167, 155	Alizarin
**6**	30.9	255.0304	0	227, 183, 171, 143, 129, 101	Purpurin
**7**	34.32	253.145	0.5	225, 209, 195	Rubiadin
**8**	34.37	269.0469	0	241, 225, 197, 182	
